# Tunable-Deformed Graphene Layers for Actuation

**DOI:** 10.3389/fchem.2019.00725

**Published:** 2019-11-08

**Authors:** Jiaqi Wang, Yukun Xiao, Volkan Cecen, Changxiang Shao, Yang Zhao, Liangti Qu

**Affiliations:** ^1^Key Laboratory of Photoelectronic/Electrophotonic Conversion Materials, Key Laboratory of Cluster Science, Ministry of Education of China, School of Chemistry, Beijing Institute of Technology, Beijing, China; ^2^Department of Biomedical Engineering, University of Michigan, Ann Arbor, MI, United States; ^3^Key Laboratory for Advanced Materials Processing Technology, Ministry of Education of China, Beijing, China; ^4^State Key Laboratory of Tribology, Department of Mechanical Engineering, Department of Chemistry, Tsinghua University, Beijing, China

**Keywords:** graphene, actuation application, structural regulation, surface modification, environmental stimulus

## Abstract

Benefiting from unique planar structure, high flexibility, splendid thermal, and electric properties; graphene as a crucial component has been widely applied into smart materials and multi-stimulus responsive actuators. Moreover, graphene with easy processing and modification features can be decorated with various functional groups through covalent or non-covalent bonds, which is promising in the conversion of environmental energy from single and/or multi-stimuli, to mechanical energy. In this review, we present the actuating behaviors of graphene, regulated by chemical bonds or intermolecular forces under multi-stimuli and summarize the recent advances on account of the unique nanostructures in various actuation circumstances such as thermal, humidity, electrochemical, electro-/photo-thermal, and other stimuli.

## Introduction

Since the successful stripping of single-layer graphene by Geim and coworkers in 2004 (Novoselov et al., 2004), this unique two-dimensional carbon material has attracted considerable attention in the energy-related fields of energy storage and conversion, advanced electronic devices, biochemistry materials, and sensors. Graphene nanolayers in particular, are ideal active components in smart actuation applications because of their intrinsic properties such as excellent transparency, superior electron conductivity and mobility (>2 × 10^5^ cm^2^ V^−1^ s^−1^ at an electron density of 2 × 10^11^ cm^−2^), large specific surface area (>2,500 m^2^ g^−1^), huge Young′s modulus (>0.5−1 TPa), and high thermal conductivity (over 3,000 W mK^−1^) (Liu et al., 2007; Moser et al., 2007; Lee et al., 2008; Morozov et al., 2008; Nair et al., 2008; Balandin, 2011; Mayorov et al., 2011; Novoselov et al., 2012). Up to now, great efforts have been devoted to the furtherance of smart graphene-based actuators by regulating the surface chemical and physical properties of graphene, leading to promising applications in robotics, sensors, mechanical instruments, microscopy tips, switches and memory chips (Osada et al., 1992; Baughman et al., 1999; Kim and Lieber, 1999; Fennimore et al., 2003; Ahir and Terentjev, 2005; Sidorenko et al., 2007; Jang et al., 2008; Park et al., 2010).

**Figure 1:**
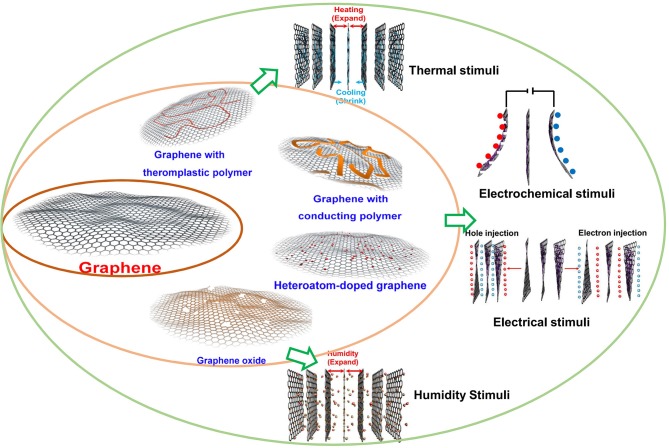
Schematic illustration of strategies toward tunable-deformed graphene materials.

Various graphene-based active responsive materials modified with functional groups via covalent bonds or non-covalent bonds have been developed recently to achieve the conversion of one or more environmental stimuli, such as electrical, light, thermal, or chemical energy to mechanical energy (Cheng et al., 2017). It is necessary and crucial to excavate and summarize the essence of actuation behaviors of graphene-based functional materials, which has not been targeted in a report yet. In this review, we focus on the deformation behaviors of graphene's internal structure modified with chemical bonds and intermolecular forces (Figure 1), and summarize the recent developments of typical important fabrication methods for the deformation of graphene sheets within the fast-growing smart fields. The state-of-art applications of graphene-based actuators will also be presented, as well as conclusions and corresponding perspectives.

## The Deformation of Graphene by Chemical Modification

It is the particular structure of graphene, an individual two-dimensional graphite composed of hexagonally network of *sp*^2^ carbon atoms that determine their unique properties, including high thermal/electrical conductivities, good light transparency, mechanical strength and flexibility. Most graphene actuation behaviors come from its inherent characteristics. For example, the electrochemical graphene actuator is mainly driven by electrical charging and discharging (Liu et al., 2012); the high photo-thermal conversion ability endows graphene with a promising light responsive component. However, the defect-free monolayer graphene shows very poor and negligible responsive behavior because of its perfect two-dimensional carbon-carbon structure with no energy gap in the electronic spectra, strong hydrophobic nature, and inert chemical reactivity, which hinders graphene from practical and widespread actuation applications. Nowadays, regulation of the carbon skeleton structure with other elements such as oxygen, nitrogen, sulfur, and phosphorus via a chemical method is an efficient way to change its *sp*^2^ bonded state and the location density of the electron cloud, in order to open the band gap and to increase the chemical active sites.

### Oxygen-Carbon Bond in Graphene

Introducing the oxygen element into the carbon-carbon structure is an efficient way to adjust the physical and chemical environment of graphene. In this regard, graphene oxide (GO) is recognized as the graphene covalently bonded with carboxyl, hydroxyl, and epoxy groups. Typically, GO sheets are synthesized by Hummer's method, in which graphite is oxidized using strong oxidants such as KMnO_4_, KClO_3_, and NaNO_3_ in the presence of nitric acid or its mixture with sulfuric acid (Kim and Lieber, 1999). The incorporation of these oxygen-related groups could change the intrinsic configurations of graphite sheets and induce the high polarization of electron density. The oxygen-containing groups bond with the carbon skeleton and distorts the structure of graphene from *sp*^2^ to *sp*^3^ hybridized states. The interlayer separation in graphene is about 0.34 nm while the inter sheet distance for GO varies from 0.63 to 1.2 nm, depending on the environmental relative humidity.

Compared with initial graphene, the existing oxygen groups in the GO system endow it with new features in advanced actuation fields. The oxygen-carbon bonds of GO nanosheets can form hydrogen bonds with water molecules in the surrounding environment, which induces the volume changes of GO materials under the heat or humidity stimuli. This hydrophilic property also causes the fast growing coefficient of thermal expansion in GO (130.14 × 10^−6^ K^−1^) compared to graphene (7 × 10^−6^ K^−1^), further enhancing the contraction amplitude as the temperature increases (Kelly, 1972; Kim and Lieber, 1999; Schniepp et al., 2006; McAllister et al., 2007).

**Figure 2:**
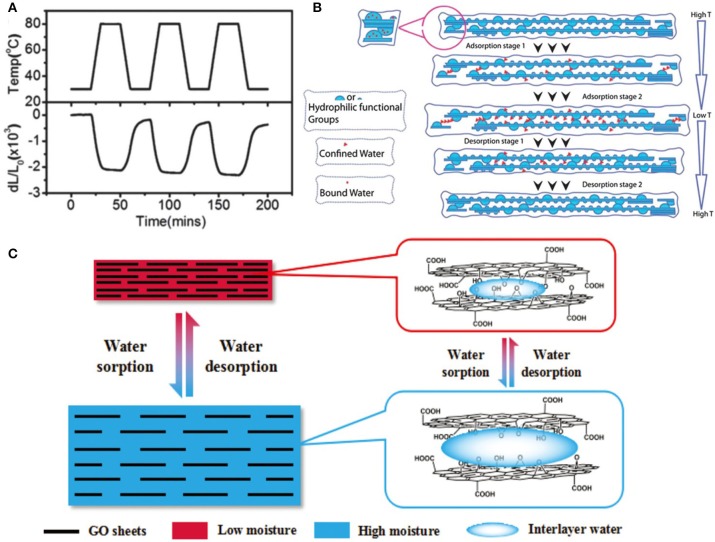
**(A)** Thermal behavior of GO paper for repeated cooling and heating cycles between 30°C and 80°C. **(B)** The process of the water adsorption/desorption process in GO paper (reprinted with permission from Zhu et al. (2012). Copyright 2012 American Chemical Society). **(C)** The process of absorption/desorption of water molecules in GO sheet (reproduced from Qiu et al. (2018) with permission from The Royal Society of Chemistry).

Zhu's group confirmed this and found that the GO paper was able to present a reversible contraction/expansion with the stimulation of heating-cooling between 30 and 80°C (Zhu et al., 2012; Figure 2A). Unlike graphene, large negative coefficients of thermal expansion in GO is derived from the change of the water molecule between the GO sheet interlayers. With abundant oxygen-containing groups, temperature and humidity changes can induce the volume expansion or contraction of GO sheets because of the absorption or desorption of water molecules between its interlayers (Zhu et al., 2012; Figure 2B). During the adsorption (cooling) stage, water molecules percolated to the protruding islands in the hydrophilic regions and increased the overall interlayer spacing. The fast saturation of these hydrophilic regions and other areas with water caused GO sheets to slide apart from each other, resulting in the elongation of the GO sheets. On the contrary, the GO sheets will contract with the collapse of interlayer spacing in the desorption (heating) process. In addition, with the oxygen atoms of these groups via hydrogen bonding, GO systems can also show an actuation response under humidity stimuli due to their ability of fast absorption/desorption of water molecules. These water molecules could act as spacers for GO interlayers, and the distance between GO sheets can be regulated from 6 to 12 Å by controlling relative humidity (RH) from high water content to low content, therefore causing the deformation of GO materials (Zhu et al., 2012; Figure 2C). Cheng et al. discovered a similar elongation-contraction phenomenon in a rotational twisted GO fiber. The twisted GO fiber could rotate fast and reversibly once exposed to a humid environment. This is attributed to the strong expansion/contraction of GO layers, adsorbing and desorbing the water molecules. The twisted GO fiber can reach a large deformation of 5% (Cheng et al., 2013). Furthermore, a kind of simple focused-sun-light-induced photo reduction method has been developed to prepare reduced rGO and GO mixed bilayer paper by adjusting the sunlight radiation intensity (Han et al., 2015). The anisotropic GO/rGO showed bending curvatures from 0° to 168° under the moisture stimulus because of different water molecules absorption ability for GO and rGO layers. Overall, the unique performance of the oxygen-carbon bonds in graphene holds great promise for advanced graphene-based smart artificial electronic devices.

### Heteroatom-Carbon Bond in Graphene

**Figure 3:**
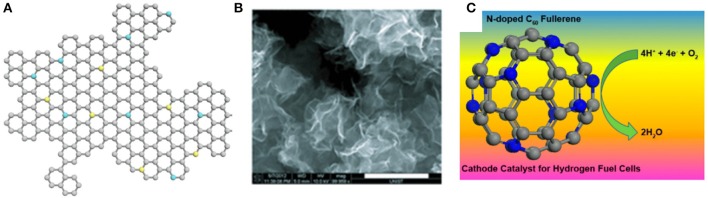
**(A)** Heteroatom doped graphene. The balls colored gray, blue, and yellow are C, O, N atoms, respectively. **(B)** SEM image of BCN-graphene. Scale bar is 1 mm (reprinted with permission from Jung et al. (2014). Copyright 2014 Wiley-VCH). **(C)** Schematic electrochemical pathway of nitrogen-doped carbon materials (reproduced with permission. Copyright 2013, American Chemical Society).

Apart from the oxygen-carbon bonding control in graphene, doping graphene with substituent heteroatoms is another efficient route to tune the electrical and chemical properties of graphene. Previous achievements have demonstrated that the graphene doped with nitrogen (N), boron (B), sulfur (S), phosphorus (P), and iodine (I) could efficiently create a disordered surface topography and modulates local surface and electric features of the conjugated carbon-carbon structures (Gong et al., 2009; Qu et al., 2010; Gao et al., 2013; Li et al., 2014; Cui et al., 2017; Figures 3A,B). Compared with the C atom, the electron-deficient B atom induces the charge polarization of the graphene basal plane. The doping of the B atom in graphene usually takes the place of the C atom on the plane to form the stable BC_3_ structure and/or out of the plane to create boric esters. While the N and P atoms are electron-rich donors, in which the P atom has an extra 3p orbital and larger atom radium, making it possible to increase the transformation of *sp*^2^ C to *sp*^3^ C (Cui et al., 2017).

It has been validated that graphene nanolayers possess electrostriction effects when injecting electrons into the carbon planar structure under the bias voltage, showing the electrochemical actuating behavior. Efficient heteroatoms-doping to graphene layers would increase the electron–ion activity and facilitate charge transfer at the electrode-electrolyte interface of graphene in the electrochemical process, facilitating the charge injection and C-C bond expansion of the graphene sheet at the interface. More importantly, the electrochemical responsive amplitude can be easily modulated by controlling the different heteroatoms doping or designing asymmetric doping in the graphene structure. In this regard, we experimentally verified the effect of different surface elemental doping on the graphene actuation ability, by constructing an asymmetrical surface modified graphene film (Xie et al., 2010). Two opposite sides of the graphene film were chemically treated with the hexane and O_2_ plasma, respectively. The side doped with oxygen groups exhibited a higher charge accumulation ability in the electrode-electrolyte interface than the other side, leading to the deformation of the graphene film.

At present, various methods have been developed to fabricate the heteroatoms doped graphene materials in the energy conversion field. In particular, the thermal annealing method is usually applied to fabricate heteroatom-doped graphene structures. Most of the precursors, such as B_2_O_3_, boric acid, urea, melamine, dicyanamide, aminoterephthalic acid, and hexachlorocyclotriphosphazene, are easily decomposed in a high temperature thermal annealing process, which are ideal candidates for constructing a single-/dual-heteroatom doped graphene (Sheng et al., 2011; Wang et al., 2012; Li et al., 2013; Fang et al., 2014; Vikkisk et al., 2014; Dong et al., 2015; Haque et al., 2015). In addition to the above method, other important routes have also been developed for the preparation of heteroatom-doped graphene, including discharge, plasma treatment, ball milling, chemical vapor deposition (CVD), and solvothermal reaction methods (Li et al., 2010; Qu et al., 2010; Deng et al., 2011; Jeong et al., 2011; Jeon et al., 2012; Dey et al., 2014; Jung et al., 2014; Hassani et al., 2016). These fabricating methods provide promising possibilities for the surface modification of graphene actuators, which are beneficial for improving the electrochemical performance in advanced energy conversion devices.

However, the exact actuation mechanism of the graphene actuator is still unclear, and requires further systematic studies focused on the electromechanical behaviors of graphene with surface modifications, leading to accurate control of the motion of the graphene actuator (Figure 3C). Nevertheless, there is no doubt that the graphene-based actuator holds great potential for applications in various electric/electrochemical responsive systems.

## The Deformation of Graphene by Intermolecular Interactions

**Figure 4:**
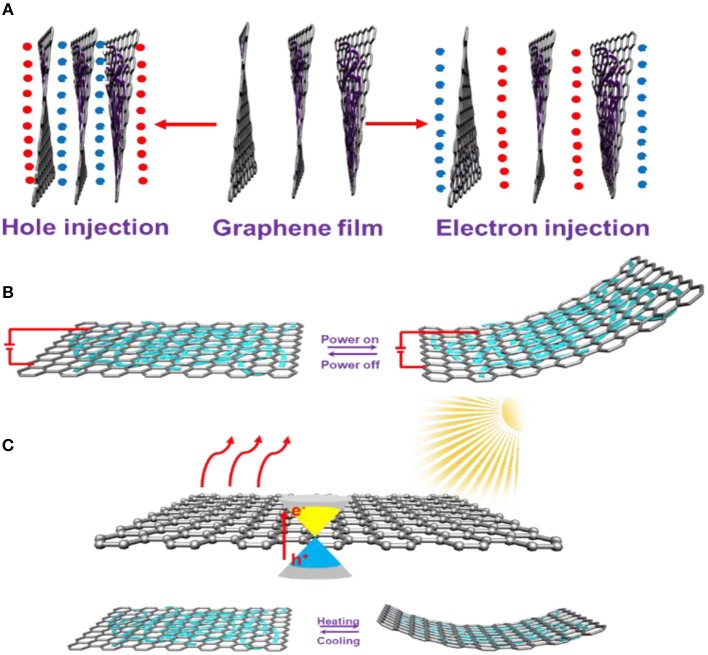
**(A)** Schematic illustration of electrons charge or discharge into the graphene films. **(B)** The stretching or bending behaviors of graphene under the electrical stimulus. **(C)** The conversion of kinetic vibration energy to heat motion energy through infrared-phonon interactions.

The unique properties of high electric-/photo-thermal and electrostriction effects make graphene an attractive active component for mechanical responsive devices (Novoselov et al., 2004, 2012; Stankovich et al., 2006). Apart from the chemical modification, graphene, especially for graphene oxide with a large 2D conjugated structure and rich oxygen-related groups, is easily combined with conjugated polymers, organic molecules, and inorganic constituents through intermolecular interactions, such as electrostatic interaction, hydrophobic interaction, chemisorption, π-π stacking, hydrogen bonding and so on. Effective contact of the active components directly determines the astute response, actuating ability, and the cycling life. Many efforts have been devoted to the combination of graphene and polymer, organic and inorganic constituents, showing great potential in energy-mechanical conversion applications (Stankovich et al., 2006; Compton and Nguyen, 2010; Dai, 2012; Cui et al., 2016; Azadmanjiri et al., 2018; Benzigar et al., 2018). In general, there are three typical responsive mechanisms for graphene-based functional composite actuators, including an electrostrictive response, electrothermal response, and a photothermal response (Figure 4).

As mentioned above, the expansion/contraction of the graphene sheet, caused by injecting electrons/holes into the carbon-carbon skeleton, can be regulated by chemical modifications. Similarly, this actuation behaviors (direction, curvature/displacement) of graphene, driven by electrical charging and discharging, can be further enhanced or precisely controlled through rational design of the graphene and active responsive components (Figure 4A). In order to achieve a large range of actuation displacement of graphene with good controllability, conducting polymers [such as polyaniline (PANI) and polypyrrole (PPy)] show the ability to convert electrical energy into mechanical energy in the electrochemical process, and are promising candidates for advanced actuators. In this case, we successfully constructed an asymmetric bilayer actuator by combining graphene and anions doped PPy in an ectropolymerization process (Liu et al., 2012; Cheng et al., 2017; Rasouli et al., 2018). Unlike the graphene deformation that operates by electron/hole injection, PPy deformation is mainly driven by the Faradaic doping and undoping process, in which the volume of the PPy structure increases or decreases with the embedding or expelling of the anions under negative or positive voltage. The discrepancy in the actuation mechanism of graphene and PPy would optimize the actuator configuration, leading to a larger bending ability than that of the graphene and PPy film itself. Such an asymmetric bilayer design can be structurally regulated into various actuation systems, including a 1D fiber actuator and a 3D framework actuator (Liu et al., 2012; Qiu et al., 2018), providing promising possibilities for the advanced actuation system.

Alternatively, as graphene has various stretching and bending vibrations in the perfect sp^2^-bonded carbon network, an appropriate electric current or infrared light applied to a graphene structure would induce Joule heating that generates the electric current passing through the graphene plane or induced the enhanced disordering degree by infrared–phonon interactions during the forced resonance vibration process, leading to the conversion of kinetic vibration energy to heat motion energy (Figures 4B,C). The higher electric power or light intensity inputs, the more thermal energy is converted. This outstanding electric-/photo-thermal conversion ability makes graphene the active component in various electric-/photo-stimuli actuators. Most thermoplastic polymers, such as polystyrene (PS), polyethyleneterephthalate (PET), poly (methyl methacrylate) (PMMA), polydimethylsiloxane (PDMS), and polyurethane (TPU), possess very weak photo- and electric-thermal conversion capacity, such that the generated thermal energy is not enough to convert into visible mechanical kinetic energy. Therefore, it is of practical significance for high-performance conventional polymer actuators to integrate with graphene sheets (Yan et al., 2010; Shi et al., 2015; Azadmanjiri et al., 2018). The functional groups of polymers can cross-link the carbon skeleton or oxygen-containing functional groups (epoxy, hydroxyl, carbonyl, and carboxyl) of the graphene or its derivatives through π-π stacking, electrostatic interaction, and hydrogen bonding. Consequently, multi-functional actuation devices are possibly designed and developed by a combination of specific polymers and graphene sheets or the structural regulation of graphene. Take the 3D graphene aerogel structure as an example, the spongy graphene aerogel with a special structure of a large area of folded material shows a larger negative coefficient of thermal expansion around −10^−4^ per °C at low voltage compared to other structured graphene materials, which makes achieving bimorph and stimulus-responsive actuators possible, by coupling with polymers.

## The State-of-Art Applications

The covalent and non-covalent functionalized graphene sheet endows it with biomimetic organism behaviors, which can achieve specific complex actions by combining functional responsive molecules or through a systematic design. These deformable actions are usually operated and controlled by external stimuli including thermal, light, moisture, and electric/electrochemistry; promising in applications ranging from sensors, switches, and artificial muscles to nano/micro electromechanical systems.

### Temperature Stimuli

Considering the fact that graphene contracts upon heating because of the distinctive large negative coefficient of thermal expansion (Bao et al., 2009; Grigoriadis et al., 2010), thermal-induced actuators based on graphene can be realized by constructing asymmetric thermomechanical responsive structures (Zhao et al., 2013). For instance, a temperature-responsive GO film was fabricated by controlling the asymmetric microporous structures in two sides of the film (Cheng et al., 2016). The GO film exhibits reversible and quick bending upon heat induced by intermittent irradiation of infrared light. To further demonstrate the potential functions, laser writing circuits were *in situ* inserted into the GO film to detect its actuation behavior in real time, leading to an integrated self-detecting sensor.

The greater the difference of deformation properties, the higher the sensitivity to the temperature stimulus. In order to enhance deformation ability, Xu et al. applied small GO sheets (the size is ~1 μm) with rich edge functional oxygen-containing groups to obtain a maximum negative coefficient of thermal expansion of the GO sheets (Xu et al., 2017). After combining with a thermoplastic poly(vinylidene fluoride) (PVDF) film, the developed bilayer actuator possessed rapid and sensitive responses with excellent stability and repeatability. In addition, the photo-/electro-thermal conversion effects of the GO were applied to various mechanical responsive actuators. Zhu et al. (2011) demonstrated a sensitive heat-induced bending cantilever by coating the CVD grown graphene on a thin epoxy cantilever. The deflection of the graphene–epoxy hybrid cantilever could increase linearly with effective conversion factors of 0.17 μm °C^−1^ and 2.58 μm mW^−1^. A large displacement of 15 mm and high displacement-to-length ratio of ca. 0.79 could be achieved by constructing a bimorph actuator with PDMS with graphene as the raw material (Chen et al., 2011), in which a curvature was about 1.2 cm^−1^, with a strain of 0.41% at 10V for 3 s.

### Humidity Stimuli

**Figure 5:**
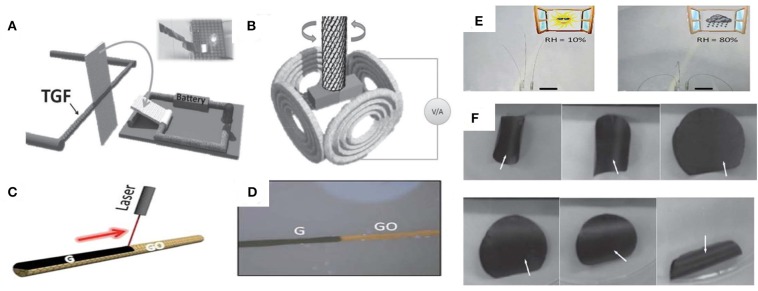
**(A)** The scheme of the designed humidity switch based on the humidity-responsive the twisted GO fiber in response to moisture (e.g., RH = 85%). **(B)** The alternating current generator. contains four copper coils around the TGF with a magnet. When the environment humidity changes, TGF can reversibly rotate the magnet within the surrounding copper coils to generate electricity (reprinted with permission from Cheng et al., 2014. Copyright 2014 Wiley-VCH). **(C)** Left: The scheme of laser positioning reduction on one side of a GO fiber. The black region corresponded to laser induced graphene region along the brown GO fiber. Right: optical image of the top surface for the as-prepared asymmetric G/GO fiber. **(D)** Photographs of a graphene/GO fiber (2 cm in length) under different RH values. (Reprinted with permission from Cheng et al., 2013. Copyright 2013 Wiley-VCH). **(E)** Photographs of three-wire moisture tentacles of the graphene/GO fibers at the sunny and rainy days, respectively. Scale bars: 5 mm. **(F)** Actuation of the bilayer paper sample as a function of RH. From left to right, RH was 12, 25, 49, 61, 70, and 90%, respectively. White-arrowed side: surface of the graphene oxide layer. (Reprinted with permission from Park et al., 2010. Copyright 2010 Wiley-VCH).

The hydration actuation behavior of GO materials induced by the strong hydrogen bond interaction with water molecules provides processing opportunities for fabricating new types of GO actuators responsive to changes in environmental water amounts and/or relative humidity (Zhao et al., 2013). The energy sources that triggers this behavior can be water vapor or thermal stimuli in an open system. In this regard, various humidity-based actuation micro-devices including robot, gripper, and electric generators have been developed recently. Take the GO sheets as an example, a kind of designed moisture-driven rotational motor was achieved by simply rotating the freshly spun GO fiber to form the twisted GO fiber (Cheng et al., 2014). This twisted GO exhibited a remarkable rotary speed up to 5,190 revolutions per min and a tensile expansion of 4.7% under humidity alternation (Figures 5A,B), which provides a way of building the moisture switches and moisture-triggered devices that convert mechanical-to-electric energy. By elaborately constructing an asymmetric structure, the actuation behaviors will be transmitted into various GO assemblies from 1D to 3D architectures. A rGO/GO asymmetric fiber actuator was fabricated in virtue of the laser positioning reduction along the one side of GO fiber, to perform bending/unbending actuation once it is exposed to humidity (Cheng et al., 2013, Figure 5C). To demonstrate the functions of the actuator, the asymmetric rGO/GO structure on the GO fiber was further regional-designed processed by a laser direct writing strategy. Consequently, the developed rGO/GO fiber actuator displayed sophisticated, programmed motions such as reversible multi-folding, S-shaped torsion, and spiral deformations (Figures 5D,E), which plays a vital role in single-fiber walking robots and shape-memory devices.

Along with this asymmetric moisture inserting strategy, more stimulus-induced deformation systems based on bimorph actuators have recently been reported (Sun G. et al., 2011; Sun Y. et al., 2011; Ji et al., 2014; Han et al., 2015; Kim et al., 2015). Park et al. (2010) demonstrated a bilayer actuator based on multi-walled carbon nanotubes (MWCNTs) and GO paper through sequential filtration method. At the initial state, the amount of water in the GO layer and MWCNT layer in the ambient is 17 and 0%, respectively. Differences in water adsorption capacity of MWCNTs and GO, leads to an interesting phenomenon: the bilayer paper will be rolled up at RH 12% of the room temperature because of the faster water absorption of MWCNTs compared to the GO side. In the meantime, it gradually becomes flat as the RH increases by 55%, due to the balance of the water absorption achieved by two sides of the film. This asymmetric paper will roll up again with the RH at 60–85% because of the large water absorption for the GO side (Figure 5F). It is noted that the differences in water molecular adsorptive capability in an asymmetric structure is essential in highly sensitive and advanced humidity-driven actuators.

### Electrical/Electrochemical Stimuli

**Figure 6:**
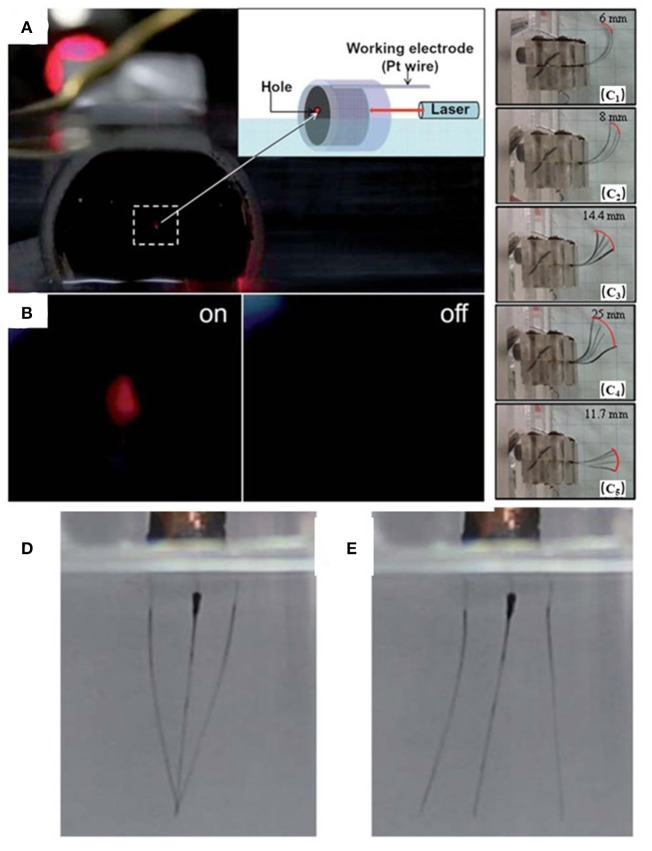
**(A)** Photograph of a designed device in which G–PPy was filled into a plastic pipeline with a microsized hole in the center of G–PPy. The G–PPy was partially immersed in 1 M NaClO4 solution and the hole was exposed to a laser beam for visibility. A Pt wire in contact with the sample was used as working electrode. **(B)** The open (on) and closed (off) state of the hole under the applied potential of +0.8 V and −0.8 V, respectively (reproduced from Liu et al., 2012 with permission from The Royal Society of Chemistry). The corresponding overlaid digital images captured at the starting point and the end point for the actuators **(C**_**1**_**)** IP -PPy, **(C**_**2**_**)** IP-PPy/rGO-1, **(C**_**3**_**)** IP-PPy/rGO-2, **(C**_**4**_**)** IP-PPy/rGO-3, and **(C**_**5**_**)** IP-PPy/rGO-4 in response to **(A,D)**. **(C)** Voltage of 6 V in amplitude (reproduced from Rasouli et al., 2018 with permission from The Royal Society of Chemistry). **(D,E)** Snapshots of the tri-armed tweezers driven by an applied electric potential of +0.8 V and −0.8 V, respectively. (Reproduced with permission. Copyright Wang Y. et al., 2013, Elsevier Ltd).

Owing to the excellent electrostriction and electro-thermal features of graphene, the responsive to electrical or electrochemical stimulations are most widely researched among other types of graphene-related stimulus-sensitive actuators. Moreover, these remarkable microscopic characteristics of graphene sheets can be transmitted into macroscopic properties of rational assembled architectures, which leads to achievements of 1D to 3D electrical/electrochemical graphene actuators for different applications. A novel 3D graphene-based actuator based on the graphene framework coated with conductive PPy was successfully fabricated by the hydrothermal method (Liu et al., 2012; Figure 6A). It exhibited a maximum strain of up to 2.5%, superior to carbon nanotube film (Xu et al., 2008), graphene film (Xie et al., 2011), and the calculated strain induced in monolayer graphene by the formation of an electrostatic double layer (Rogers and Liu, 2011). The 3D prominent composite can be used as a smart filler for a controlled laser switch by adjusting the applied voltage (Figure 6B), which was filled into a plastic pipeline with a hole left in the center of the filler in order to let the red laser spots pass through. When applying a positive potential of 0.8 V, the initial red spot is blocked by the hole contraction induced by the expansion of the composite. This on/off switch process is repeatable and reliable, and can also be durably run for several months.

Rasouli reported a PPy/rGO-based ionic actuator by simple electropolymerization of PPy/rGO nanocomposites on both faces of ink-coated Nafion membranes (Rasouli et al., 2018). When applying voltage on the composite film, the solvated ions would accumulate in a high concentration near the electrode regions, leading to bending deformations (MacDiarmid, 2001; Terasawa and Asaka, 2016). The maximum bending deformation of the actuators under the voltage of 6 V was about 25 cm compared to that of conventional ionic polymer (IP)–metal composites actuators (~23 cm, Shown et al., 2015). Except for the enormous deformation, the PPy/rGO-based ionic actuators also possessed excellent ion conductivity, capacitive characteristics, and large charge storage capacity (Figures 5C_1_–C_5_). Moreover, the electrochemical actuation behaviors are also reflected on 1D fiber actuators (Wang E. et al., 2013). A 1D fibrillar actuator based on PPy/GF was constructed by partially electropolymerizing of the PPy on the graphene fibers (GF). With a high strength of 230 MPa, a good maximum bending angle, and reasonable durability, this actuator can work as a micro tweezers (Figures 6D,E), which makes it possible to be the fiber-robot to operate multiple surgery or cell operation in the future.

**Figure 7:**
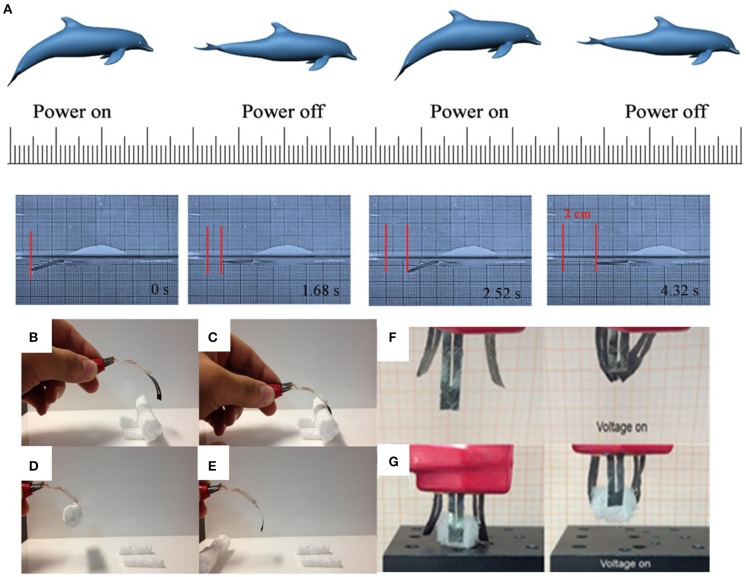
**(A)** Diagram to demonstrate the fish-like robot swimming, when the power is on or off, the “tail” bends down or up, then the fish-like robot will swim forward. The dimensions of the fish tail (the graphene-PVDF bimorph robot and the fish body (Expandable PS) are 14 × 3 mm^2^ and 30 × 8 mm^2^, respectively (reprinted with permission from Xiao et al., 2016. Copyright 2016 Wiley-VCH). **(B)** Laser reduced graphene oxide-based ETA without driving voltage. **(C)** The laser reduced graphene oxide-based electrothermal actuator is driven by the voltage of 65 V. **(D)** The LRGO-based ETA is hooking the plastic foam for moving a distance. **(E)** The driving voltage is switched off and the plastic foam is placed on the test bed (reprinted with permission from Zhang et al., 2017. Copyright 2017 Wiley-VCH). **(F)** A “tri-finger” mechanical gripper with 10 V turned off (left part) and turned on (right part). **(G)** A larger object is grabbed by the “tri-finger” gripper at applied 10 V (reproduced from Hu et al., 2014 with permission from The Royal Society of Chemistry).

Since the discovery that the single-walled carbon-nanotube incorporated into chitosan could generate electromechanical actuation properties at low alternating voltage (AC) stimuli (Hu et al., 2010), the electro-thermal graphene based actuators in the ambient solution, except for the electrical/electrochemical actuation in the solution, have also recently been widely investigated. Usually, the actuation ability is determined by Joule heating which is generated by the electric current passed through the graphene-based film. The higher the electric power input, the more thermal energy is converted. To this end, Xiao et al. reported an electromechanical actuator constituted by a porous graphene paper and PVDF layer (Xiao et al., 2016; Figure 7A). The hybrid film exhibited a large actuation motion with a maximum deflection of about 14.0 mm within 0.262 s and generated high actuation stress (>312.7 MPa g^−1^). This phenomenon is the same as that of the bilayer responsive structure of graphene and organic glass substrate which exhibited a reversible and large bending angle of 270° with a fast response of 8 s and recovery period of 19 s under the driving voltage of 65 V (Zhang et al., 2017). The difference in thermal expansion coefficient values between the thin graphene film and the organic glass substrate gives rise to an expanded volume of the two layers in different amounts, thus resulting in the deformation of the actuator (Figures 7B–E). In addition, Chen's group demonstrated an SG/PDMS bimorph actuator by combining spongy graphene (sG) paper with PDMS, which showed an ultra-large bending displacement of 15 mm with a curvature of about 1.2 cm^−1^ at 10 V for 3 s, a high displacement-to-length ratio of ~0.79, and vibration motion at AC voltage up to 10 Hz (Hu et al., 2014; Figures 7F,G), superior to those similar bimorph actuators reported previously (LeMieux et al., 2006; Sul and Yang, 2009; Hu et al., 2010; Jeon et al., 2012; Bi et al., 2013). More importantly, this bimorph actuator could mimic the fingers to fast grab, move and put down the objects, providing a basis for various device designs in the fields of artificial muscles, robotics, sensors, medicines and so on.

### Light Stimuli

**Figure 8:**
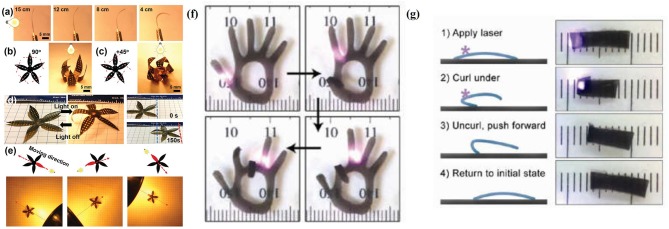
**(a)** Photographs of a PLP actuator upon exposure to light with different distances. **(b,c)** Photographs of a flower-shaped PLP actuator with **(b)** *y* = 901 and **(c)** *y* = +451 when exposed to light. **(d)** Photographs of a PLP robot crawling by switching the light on and off. **(e)** Schematic and photographs of a PLP robot with multi-way gaits under light stimulus (reproduced from Deng et al., 2018 with permission from The Royal Society of Chemistry). **(f)** Images of the fingers of a hand-shaped rGO-ELP hydrogel controllably bending and unbending in response to the location of an IR laser spot. **(g)** Schematic and images of a light-driven crawler. A rGO-ELP hydrogel molded with a slight curvature was placed with the porous side facing down. The laser was applied so as to induce gel curling. Subsequent uncurling during recovery after the laser was removed pushed the gel forward (1 mm tick marked) (reprinted with permission from Wang E. et al., 2013. Copyright 2013 American Chemical Society).

As actuation active components, graphene and its derivatives with the extraordinary conversion of kinetic vibration energy to heat energy of graphene sheets, induced by the infrared phonon strong interactions during the resonance vibration process, have been applied to infrared light-driving actuators (Zhao et al., 2013). Lin and co-workers developed multi-responsive soft actuators with laser induced graphene (LIG) patterns as geometrically constraining elements coupled with PVDF and polyimide (PI) using a direct laser writing method (Deng et al., 2018). This PVDF/LIG/PI (PLP) sandwich actuator was a programmable shape transformation, in which responsive flowers with designed shapes were realized, in order to perform different bending behaviors under the controlled irradiation of the lamp (Figures 8a–e), imitating bionic robots. Another example is the combination of rGO and elastin-like polypeptides (ELPs) to simulate human finger actions (Wang E. et al., 2013). To demonstrate their idea, a hand-shaped rGO–ELP gel was fabricated, in which these “fingers” performed the given bending movement by controlling the irradiation position (Figure 8f). The bending rate and angle of the finger increased as enhancing the infrared radiation laser intensity and rGO concentration. Moreover, the rGO–ELP actuator can also be made into a light-driven crawler to “walk” on glass slides under the light radiation (Figure 8g). When applying infrared radiation on the one side of the crawler, it folded immediately. Once the light is off, the front rose and the back of the crawler produced a forward-directed force by pushing against the glass as it uncurled, which moved the entire gel forward 3 mm. These light-stimulated actuation strategies provide material platforms for the advanced smart muscles, robotics, and intelligent sensors. Recently, Wang et al., successfully prepared PDMS/graphene composite bilayer film, which could bend 7.9 mm in the horizontal direction under the light radiation. Moreover, this bilayer actuator can be further constructed into a beluga whale soft robot to swim in the pool at the speed of 6 mm/s deriving from its superhydrophobicity (Wang et al., 2019).

## Conclusion and Outlook

The actuators with the graphene as active components show great potential in the various intelligent bionic devices. Particularly, the actuating behaviors of graphene regulated by chemical bonds or intermolecular forces under multi-stimulus are discussed, which endow graphene-based responsive units with response capability to single and/or multiple environment stimulus, including electro-thermal stimuli, humidity stimuli, temperature stimuli and so on. For instance, one way is to selectively decorate the graphene with heteroatoms, such as oxygen, in which graphene oxide is fast and sensitive to water molecules, showing excellent responsive behavior to humidity. The other way is to specifically modify the graphene with conducting polymers or thermoplastic polymer, making it a crucial part of smart switch system or intelligent robotics. These prominent features present promising applications in smart systems with complex functions.

However, there are still some challenges that remain to be solved to meet future requests of graphene-based intelligent materials. The high manufacturing cost and delayed response time of graphene-based actuator have always been restricting the practice of advanced application in industry and intelligent home system. It is still an efficient way to introduce highly sensitive responsive polymers or gels into graphene systems to construct high performance bionic intelligent systems, such as modified PDMS, PMMA and other new designed organic polymers. Moreover, the mechanical properties and durability of the current graphene-based actuation system needs to be promoted, which involves preparation of high-quality graphene, matching of graphene with functional materials, and the design of specific graphene microstructures or assemblies. In addition, it is highly desirable to constitute smart graphene systems that handle multi-stimuli, have good sensitivity and high efficiency. Considering the promising potential application of bionic intelligent systems in the future, low cost biocompatible graphene-based actuators are also in great need of development, which remains a big challenge to overcome in the production process. There is no doubt that graphene-based smart devices will soon be playing an important role in modern intelligent systems, with various possibilities and continuous research efforts.

## Author Contributions

JW and YX are responsible for the main text. VC provided the adjustment suggestions. CS was responsible for drawing some figures. LQ and YZ hold the overall ideas and corrections.

### Conflict of Interest

The authors declare that the research was conducted in the absence of any commercial or financial relationships that could be construed as a potential conflict of interest.
